# Temperature Effects on Force and Actin–Myosin Interaction in Muscle: A Look Back on Some Experimental Findings

**DOI:** 10.3390/ijms19051538

**Published:** 2018-05-22

**Authors:** K. W. Ranatunga

**Affiliations:** School of Physiology, Pharmacology & Neuroscience, University of Bristol, Bristol BS8 1TD, UK; K.W.Ranatunga@Bristol.ac.uk; Tel.: +44-117-931-1969

**Keywords:** muscle force, muscle shortening, crossbridge force, temperature-sensitivity, actin–myosin, crossbridge cycle, endothermic force

## Abstract

Observations made in temperature studies on mammalian muscle during force development, shortening, and lengthening, are re-examined. The isometric force in active muscle goes up substantially on warming from less than 10 °C to temperatures closer to physiological (>30 °C), and the sigmoidal temperature dependence of this force has a half-maximum at ~10 °C. During steady shortening, when force is decreased to a steady level, the sigmoidal curve is more pronounced and shifted to higher temperatures, whereas, in lengthening muscle, the curve is shifted to lower temperatures, and there is a less marked increase with temperature. Even with a small rapid temperature-jump (T-jump), force in active muscle rises in a definitive way. The rate of tension rise is slower with adenosine diphosphate (ADP) and faster with increased phosphate. Analysis showed that a T-jump enhances an early, pre-phosphate release step in the acto-myosin (crossbridge) ATPase cycle, thus inducing a force-rise. The sigmoidal dependence of steady force on temperature is due to this endothermic nature of crossbridge force generation. During shortening, the force-generating step and the ATPase cycle are accelerated, whereas during lengthening, they are inhibited. The endothermic force generation is seen in different muscle types (fast, slow, and cardiac). The underlying mechanism may involve a structural change in attached myosin heads and/or their attachments on heat absorption.

## 1. Introduction

As first proposed in the mid-1950s, and referred to as the sliding filament theory, active muscle contraction involves the relative sliding between two sets of filaments, the thin (A = actin) filaments and the thick (M = myosin) filaments in a sarcomere ([1,2] refs therein). The driving mechanical process is a repetitive interaction of myosin heads (crossbridges) on actin filaments; a crossbridge attaches to actin, undergoes a conformational change generating muscle force, and power, and then detaches. This mechanics cycle is coupled to an enzymic reaction, hydrolysis of ATP by acto-myosin ATPase [3], so that energy liberated during release of the products of ATP hydrolysis (phosphate = Pi, and adenosine diphosphate = ADP) is converted into work (and heat); an active muscle is a machine converting chemical to mechanical energy. Despite many investigations and using different techniques, exactly how various steps in these two cyclic processes, chemical and mechanical, are coupled during an active muscle contraction, remains not fully understood [4]. 

Also, as has been known for many years, muscle contractile response and function are sensitive to temperature. Experiments on isolated amphibian and mammalian skeletal muscles [5,6] and on in situ human muscle [7] clearly showed that the maximally activated muscle can operate over a wide temperature range (0–40 °C) and the maximal force is increased with warming, to almost a plateau level. Subsequent studies on mammalian muscle [8] have confirmed these observations and shown that maximal active force increases (reversibly) ~2-fold on warming from 10 °C to temperatures closer to physiological (>30 °C). However, the relationship of the results from temperature studies on muscle to findings from other studies, listed earlier, remains unclear.

The primary aim of this review is to reflect on some of the basic experimental findings in such temperature-studies on mammalian muscle. Emphasis is on observations rather than detailed quantitative analyses, interpretations, and predictions. It is seen that the basic sliding filament theory and crossbridge theory are appropriate in dealing with muscle contraction at a wide range of temperatures. Analyses of force responses to a rapid increase of temperature (T-jump) show that crossbridge force generation is endothermic (i.e., is enhanced by heat absorption) and is coupled to an early kinetic step in the crossbridge cycle, probably prior to the release of inorganic phosphate (Pi). The sigmoidal temperature dependence of steady active force is mainly due to the endothermic nature of the force generation step. This step is strain-sensitive, enhanced by negative strain and inhibited by positive strain, perhaps providing a basis for increased energy consumption in shortening contraction-producing power. A possible structural mechanism for endothermic force generation in an attached crossbridge may well be a “protein folding/unfolding-process” within an attached crossbridge (myosin head) [9], or outside [4], or both.

## 2. Materials, Methods, and Techniques

This review describes complementary results from two muscle fibre preparations, namely electrically activated intact fibre bundles (from fast and slow muscles of rat) and chemically activated skinned fibres (from rabbit and rat). Only some essentials of materials and methods, relevant to temperature studies, are given here; comprehensive details can be found in the original publications.

### 2.1. The Recording System

A trough system was mounted on an optical microscope stage. In later studies, it comprised three ~50 μL troughs machined from a titanium block and a front specimen trough built with glass windows in the front and at the bottom. The temperature of the whole trough system was kept <5 °C, but, by using Peltier modules, the specimen trough temperature could be independently clamped as required. The temperature sensitivity of the force (i.e., tension) recording was reduced by using a transducer with two AE 801 elements (Akers, Horten, Norway). One cut-beam element was linked to the muscle fibre (natural resonant frequency ~14 kHz), and the other element nearby acted as a dummy, forming a full bridge exposed to the same temperature [10,11]. A custom-built moving-coil motor generated ramp and step changes in muscle fibre length.

### 2.2. The Temperature-Jump Protocol

A pulse from a Nd-YAG laser (Schwartz Electro-Optics Inc., Orlando, FL, USA) induced a T-jump in the front specimen trough (laser wavelength was near infra-red (λ = 1.32 μm), pulse power = 2 J maximum and width 200 μs). The energy absorption by water at this wavelength (~50%) was such that the laser pulse that entered the trough through the front window was reflected back by the aluminium foil at the back wall, and this raised the temperature of the 50 μL aqueous medium in the trough together with the fibre immersed in it, uniformly by 3–5 degrees in 200 μs. The higher temperature around fibre remained constant for ~0.5 s, but Peltier temperature-clamping could be used to increase this time [10].

### 2.3. Experiments

Details of the experimental procedures are given elsewhere (Ranatunga, 2010, [12]). Briefly, an intact muscle preparation was superfused with physiological saline solution containing (mM) NaCl, 109; KCl, 5; MgCl_2_, 1; CaCl_2_, 4; NaHCO_3_, 24; NaH_2_PO_4_, 1; sodium pyruvate, 10 and 200 mg·L^−1^ of bovine foetal serum. The solution was aerated with a 95% O_2_ and 5% CO_2_ mixture (pH was 7.6–7.2 at temperatures 10–35 °C, [8]). A muscle (fibre bundle) was stimulated with supramaximal voltage pulses (<0.5 ms duration) applied to two platinum plates on either side. For skinned fibre experiment, a segment of a single fibre (2–4 mm long) was glued between two hooks using nitrocellulose. The buffer solutions contained 10 mM glycerol-2-phosphate (a low temperature-sensitive pH buffer) and also 4% dextran (mol wt ~500 kDa) to restore the inter-filament lattice spacing to normal dimensions. The ionic strength was = 200 mM, pH = 7.1 and the major anion was acetate. For other details of the solution compositions, see [12,13]. The sarcomere length in a 0.5 mm length of fibre close to the tension transducer was followed using diffracted beams from a He-Ne laser. The sarcomere length was set at ~2.5 μm.

### 2.4. Some General Comments

When analysing tension responses (force transients) generated by different rapid perturbations on a muscle, it is necessary to identify the homologous components. Consistent with the terms used in our previous papers [13,14,15], various components, or phases, can be recognised in the force transients. Phase 1 refers to the force change that occurs simultaneously with the applied perturbation. The extreme force reached at the end of the perturbation is T_1_, as described in the length step (release) experiments of Huxley & Simmons (1971, [16]). A similar drop in force (phase 1) is also evident in T-jump (or in pressure-release = P-jump) experiments. This is thought to be due to expansion of a series elasticity in the fibre. A T-jump or a P-jump also induces a drop in force in rigor muscle fibres, and this provides support for the idea that expansion in some elasticity may occur [17,18]. Following phase 1 in a length release step, the force recovers quickly to the T_2_ force level. This partial force recovery (phase 2) comprises two exponential processes [19], referred to as phase 2a (fast) and phase 2b (intermediate) [15]. The force increase, i.e., the rise in force above the pre-perturbation level, induced by a T-jump (and a P-jump) corresponds to phase 2b. In T-jump and P-jump experiments, where a substantial phase 1 was seen ([14,20]), a quicker force recovery step similar to phase 2a was also seen. This phase nearly fully restored the force to its pre-step level, as in the length release experiments. The phase 1 and phase 2a processes are not prominent in the small amplitude T-jumps described here. Phase 3 is a slower exponential recovery in the force transient induced by a T-jump (and a P-jump) in the isometric state.

In brief, phase 2b is an endothermic force rising reaction seen after a T-jump.

## 3. Some Experiments on Intact Muscle

On the basis of the characteristics of their force, three functional and mechanics states may be recognised in skeletal muscle, namely the resting (or relaxed) state, the rigor state, and the active state. Interestingly, these three mechanical states also differ with respect to temperature-effect on their tension [21]. The resting muscle tension is largely temperature-insensitive. In resting state, the crossbridges remain detached, and force develops on stretching beyond rest length; this resistance to stretch arises from stretch of non-crossbridge structures in sarcomeres, such as titin filaments that attach thick filament ends to the Z-discs in sarcomere. The resting muscle force is considered to be “rubber-like”, and increases slightly at higher temperatures. Stretched resting muscle, however, can develop a type of “active” force, due to heat-contracture at high temperatures [21,22]. In rigor, with no ATP, all crossbridges remain attached to actin, but they are not cycling; muscles are stiff, and this force decreases slightly and linearly with increase of temperature (exothermic), like in a normal elastic element (after death, muscles go into rigor). In active muscle, crossbridges are cycling, i.e., they attach to actin, develop force, and detach, and, as presented below, active force is very sensitive to temperature and rises with temperature upon absorption of heat; this process is endothermic.

### 3.1. The Length/Tension Relation

**Figure 1:**
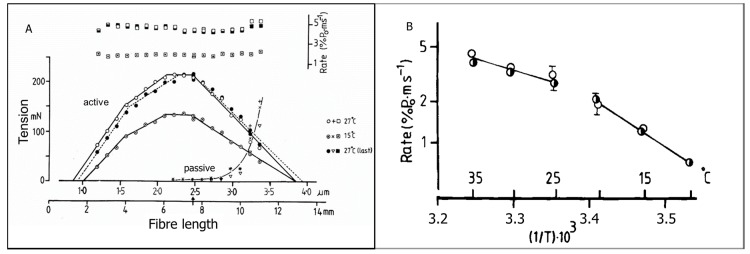
(**A**) Variation with fibre length of tetanic tension (circles) from an experiment (on rat biceps muscle, from Elmubarak & Ranatunga 1984, [24]). Data collected first at 27 °C (open circles), after cooling to 15 °C (lower set) and then after rewarming (filled circles): fibre length is shown in lower *x*-axis, and corresponding sarcomere length in the upper *x*-axis. Length/tetanic tension relation consists of four linear segments, and is reversibly lowered in cooling, but essential features remain the same. The rate of tension rise (squares, upper data) is insensitive to length, but is decreased at low temperature. At both temperatures, the passive tension in resting state (triangles, crosses) increases exponentially with length. (**B**) The rate of tension rise (mean ± SEM) from four experiments recorded at different temperatures (in cooling and warming) is plotted as an Arrhenius plot (log rate versus reciprocal absolute temperature, 1/T). The decrease in the rate with cooling is less at higher temperatures (35–25 °C, temperature coefficient (Q_10_) of ~1.5), but it is increased at the lower temperatures (Q_10_ of 2.8).

According to sliding filament theory, contractile force depends on the sarcomere length. Changes in sarcomere length change the overlap between the thick (myosin) and thin (actin filaments), and this will change the number of overlapped myosin heads able to make acto-myosin links (or crossbridges). This was established in 1966 from elegant experiments on frog muscle fibres by Gordon, Huxley & Julian [23]. The length–isometric tetanic tension relation from a small fast muscle (biceps brachii of rat forelimb), shown in Figure 1A, is basically as expected from the sliding filament theory ([24]); the length–tension relation consists of four linear regions, two forming the ascending limb. The highest tension is developed at sarcomere lengths of ~2.2–2.5 µm (i.e., at optimal filament overlap in the rat muscle sarcomere) and force is predicted to decrease to zero when the fibre is stretched to a sarcomere length of ~4.0 µm (no overlap). More importantly, this length–tension relation remains similar at high and low temperatures (27 and 15 °C) except that the tension at the full range of length is higher at the higher temperature. Thus, the basic sarcomeric structure remains the same at different temperatures. The sliding filament theory and the same crossbridge processes would likely be appropriate underlying mechanisms to consider in evaluating the contractions at different temperatures.

Figure 1B shows another finding, namely, the rate of increase in tension in isometric tetanic contractions at different temperatures. The positive peak of the differentiated tension record was measured as the maximum rate of tension rise in the tetanus. It is seen that the negative slope of the plot (rate versus 1/absolute temperature, 1/T, Arrhenius plot) is less at the higher temperature range, and is increased at the lower range. Such change of slope in rate measurements has been found in mammalian muscle contractions with an apparent transition at ~23 °C (see below and [8]). 

### 3.2. The Force–Velocity Relation

In separate experimental studies, and to determine the temperature sensitivity of the maximum velocity in shortening muscle (Vmax), force–velocity measurements were made at temperatures between 36 and 10 °C. The experiments used fibre bundles isolated from fast-(flexor digitorum longus) and slow-(soleus) twitch muscles of rat hind leg: The force–velocity relations were obtained by carrying out isotonic releases from the isometric tetanic tension plateau [25].

**Figure 2:**
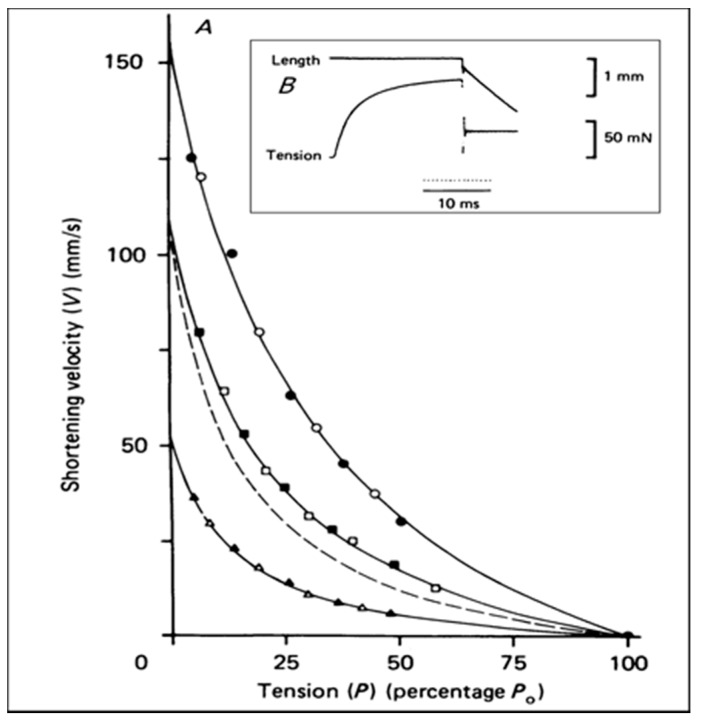
(**A**) Force-shortening velocity data at 35 °C (circles), 25 °C (squares), and 15 °C (triangles) from an experiment on a fast muscle (adapted from Ranatunga, 1984, [25]). Curves are the fitted with A. V. Hill hyperbolic equation to calculate the Vmax (velocity at zero force). Shortening velocity was measured from the slope of length record as shown for one force in (**B**), at various levels of force. The dashed line represents the force–velocity relation at 15 °C, scaled up to the maximum velocity of the 25 °C curve, to show the increased curvature at the lower temperature. The closed and open symbols are from data obtained with decreasing and increasing isotonic tensions.

Figure 2A shows a sample set of data collected from an experiment on one fast muscle at three different temperatures (35, 25 and 15 °C); the measured velocities are plotted against isotonic force levels (normalised to isometric force P_0_ at that temperature) and a Hill hyperbolic curve fitted. It is seen that Vmax (velocity at zero force) and velocities at similar relative force levels are decreased with cooling, so that the force–velocity curve is shifted down the velocity axis. It was also seen that the curvature of the force–velocity relation was increased by cooling. This is shown by the dotted curve, which is the 15 °C curve, superimposed on that at 25 °C, after normalising to Vmax at 25 °C. It is known that the curvature of the force-shortening velocity relation at each temperature is higher in slow muscle, but the increase of curvature at low temperature is seen in slow muscle, as well as fast muscle (The force-shortening velocity relation has been well studied in frog muscles (fibres) at low temperatures, but it is not clear whether the curvature of the relation changes with temperature, as in mammalian muscles).

Shortening velocity at zero force (Vmax) and at all other force levels decreased with cooling. Interestingly, when examined as Arrhenius plots (as in Figure 1B), Vmax in both fast and slow muscles showed a biphasic distribution against temperature (1/T). The Q_10_s obtained from regression analysis were 1.8 and 2.4 for fast, and 2.0 and 3.5 for slow, respectively, for the ranges 35–25 °C and 20–10 °C. It is also relevant to note that in both types of muscle, the curvature of the force–velocity relation increased at the lower temperatures, so that the mechanical power output (PxV) decreased substantially in cooling from 35 to 10 °C. As shown in a subsequent re-analysis [26], maximum power output in the two muscle types decreased to 3–5% of that at 35 °C on cooling to 10 °C. In the physiological range of temperatures (25–35 °C), the Q_10_ for maximum power was 2–2.5 in both muscles; the decrease was more pronounced below 20 °C, Q_10_ of 5–7.

Thus, as for maximum rate of tension rise in Figure 1B, Vmax and velocity near Vmax decreased less in cooling to ~25 °C, but significantly more in cooling below, indicating an apparent transition (change of slope) at ~23 °C. Indeed, similar increased temperature-sensitivity has also been made when the rates of tension relaxation were examined [8,27].

A simplistic interpretation of these observations is that the rate-determining step of a reaction, or a process, is associated with its temperature sensitivity (Arrhenius activation energy), and a transition induced by temperature changes may show a change in this rate-determining step [28,29]. The data obtained in muscle studies for rates of tension rise and relax, and for shortening velocity, are not strictly quantitative, because of the presence of an unknown amount of series (tendon) compliance in the intact muscle preparations used. Further, it is not possible to assign, to particular transitions, the temperature effects on these rates, whether in the crossbridge cycle or in the activation pathway. However, the results clearly show that one, or more, of the underlying processes in the contractile cycle in mammalian muscle undergoes an abrupt and marked change in cooling below about 22–23 °C.

**Figure 3:**
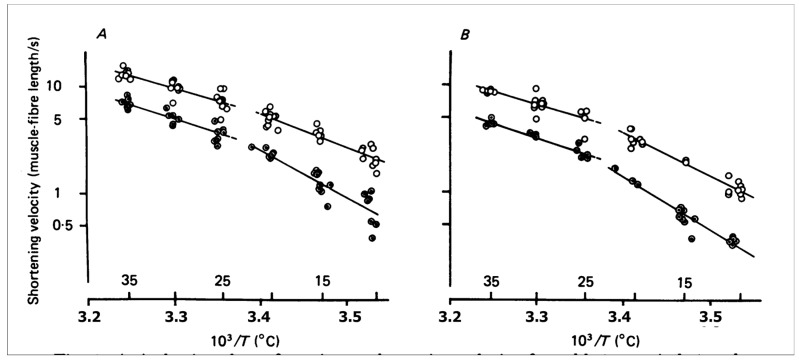
(**A**) Maximum shortening velocity for fast (open circles) and slow (circles with center dots) muscles shown as Arrhenius plots (from [25]). Pooled data are each from 14 muscles, with log velocity plotted vertically against 1/T horizontally. The straight lines are calculated regression lines for the data at temperatures above and below 23 °C. The Q_10_s obtained from the regression analysis were: fast fibres 1.8 and 2.4 and slow fibres 2.0 and 3.5, respectively, for the higher and lower temperature ranges. (**B**) Arrhenius plots for measured shortening velocity at isotonic tensions of ~10% Po from the same experiments (the presentation is similar to (**A**). The Q_10_s were 1.8 and 3.1 for fast, and 1.9 and 3.8 for slow fibres at temperatures higher and lower than 23 °C.

Biochemical studies indicate that ADP release may limit shortening velocity in muscle [30], and measurements made on myosin isoforms from a range of muscle types indicate that different shortening velocities may be due to differences in their ADP release rates [30,31,32]. Thus, the ADP release rate in crossbridge cycle may define the rate of detachment of crossbridges and increased shortening velocity, as suggested from modelling and other studies [33,34]. Interestingly, the ADP release rate from AM and the rate of ATP-induced dissociation of AM [35] have different temperature sensitivities; it is possible that the former may limit maximum shortening velocity at high temperatures (*>*23 °C), whereas the latter process, involving ATP (and hydrolysis), may be limiting velocity at lower temperatures. In principle, this can account for the change in temperature sensitivity of shortening velocity, as in Figure 3 [25].

### 3.3. Temperature Dependence of Isometric Tetanic Force

As referred to before, several studies have shown that maximum force in active muscle rises with increase of temperature, and reaches a steady level at higher temperature; results showed that the steady high level is perhaps reached at a lower temperature in frog muscle (~10 °C, [5]) than in mammalian muscle (>25 °C, [8]). Such findings initiated examination of the tetanic force at a wider temperature range in a suitable mammalian muscle preparation; the muscle used was a foot muscle from rat, flexor hallucis longus [36].

**Figure 4:**
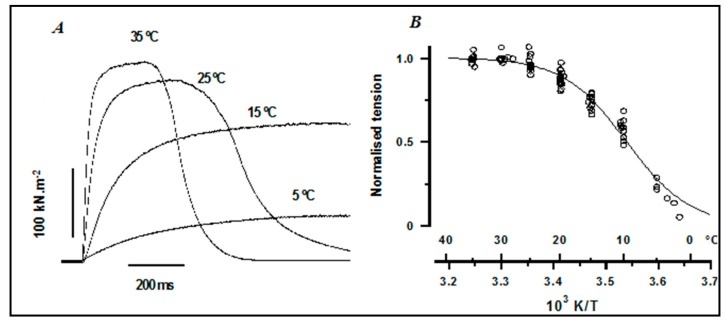
(**A**) Tetanic tension responses obtained from one intact fibre bundle at four different temperatures, using suitable stimulation frequencies and durations. (**B**) Tetanic tension data from 8 bundles at different temperatures normalised to that at 35 °C; the horizontal axis is reciprocal absolute temperature (also labelled in °C, from Coupland & Ranatunga [36]).

Figure 4A shows records of isometric tetanic contractions from an experiment on a rat muscle at a wider range of temperatures. Contractions are slower, and the force is lower at the lower temperatures. Figure 4B shows tetanic tension data collected from several such experiments; data were collected in warming and cooling. The data shows that tension increase reaches a “steady” level at physiological temperatures (>25 °C). It is found that the relation between force and (reciprocal) temperature is approximately sigmoidal, with a half-maximal tension at ~10 °C. Since the preparation was short, it was suitable for laser T-jump experiments, the first of its kind on an intact muscle.

**Figure 5:**
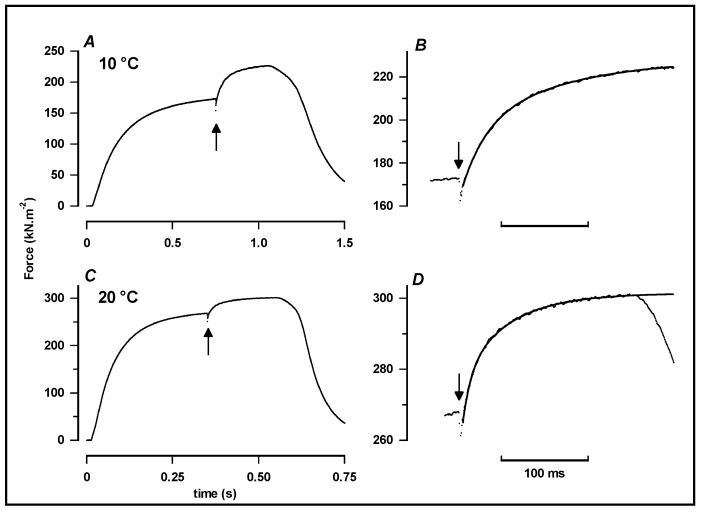
(**A**) A tetanic contraction of a fibre at 10 °C with a T-jump of ~4 °C applied at the plateau. This causes a small instantaneous tension drop (phase 1), followed by a slower rise to a new steady level. (**B**) Enlarged version of (**A**) near the T-jump. The tension response is fitted by a bi-exponential curve. (**C**,**D**) Similar plots to (**A**,**B**) from the same bundle at 20 °C: the maximum tension is higher, whereas the tension increase induced by the T-jump is faster, but smaller, than at 10 °C.

Figure 5 shows the tension response induced by a T-jump, when applied on the plateau of tetanus. As found in (maximally) Ca-activated skinned fibres [10,17,37,38], a T-jump induces a biphasic tension rise that reaches a new steady level, both at 10 and 20 °C; the initial tension rise (phase 2b) is faster but the amplitude of tension rise is smaller at the higher temperature.

A careful look at some other studies shows that, with increased temperature, (1) the muscle stiffness remains unchanged ([39] and refs therein); i.e., number of crossbridges attached is unaltered; (2) the tension/stiffness ratio is increased [40]; i.e., average force per attached crossbridge is higher and (3) from single-molecule experiments of Kawai et al. [41] the force each crossbridge generates is independent of temperature. It may be argued that development of steady active force in isometric muscle may be simplified to a two (or three) state system—one low-force (pre-force generating) state and one (or two) high force states. With increased temperature, the total number of attached crossbridges remains the same but, due to endothermic force generation, higher temperature favors high force states. In principle, such a simplistic scheme can qualitatively account for the sigmoidal temperature dependence of active force as being due to the shift in the equilibrium from low- to high-force states (Davis, [42]; Roots & Ranatunga, [39]).

### 3.4. Force During Shortening/Lengthening

**Figure 6:**
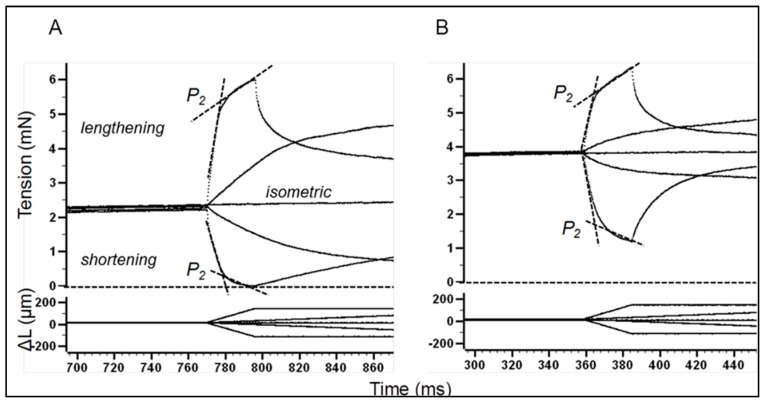
Records of tension (upper panel) and length (lower panel) from a muscle at 10 °C (**A**) and at 35 °C (**B**); isometric force (P_0_) is nearly doubled at 35 °C. At the isometric tetanus plateau, a ramp shortening or lengthening of ~6% was applied (bottom traces) at two velocities, 0.25 and 2.3 L_0_/s. The tension at the (P_2_) transition was measured (adapted from Roots et al. [43]) at the point of intersection between two lines, fitted as shown (this represents crossbridge force during shortening/lengthening). The dashed line represents zero active force level.

**Figure 7:**
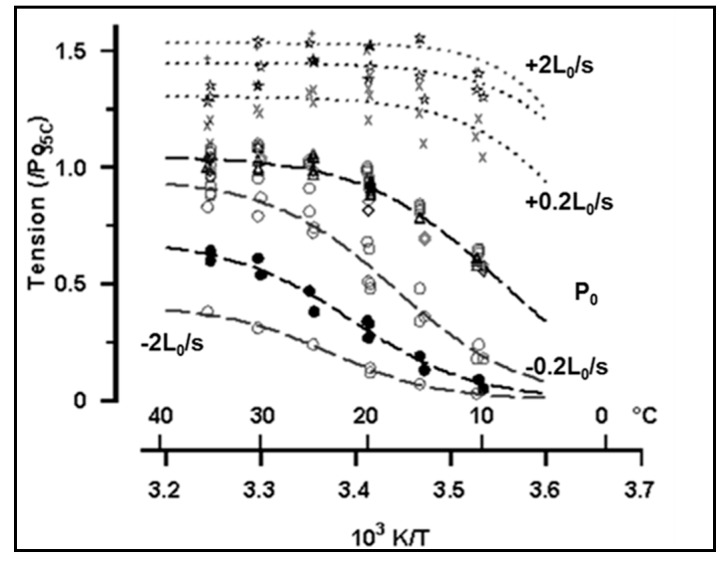
Dependence of tension on temperature during ramp lengthening and shortening. Pooled data from three fibres in which data for isometric (P_0_), shortening (−) and lengthening (+) were recorded at all the temperatures shown. Tensions were scaled to the isometric tension at 35 °C. Note that the isometric tensions P_0_ (Δ, ◊, □, *n* = 120) and P_2_ for shortening (●, ○, *n* = 8–20) at a given velocity increase with warming: the distributions are all sigmoidal. From fitted curves, temperature for half-maximal tension is ~9 °C for isometric; it increases with shortening velocity and is 23 °C for −2 L_0_/s (as first reported in [39]). The tension for each lengthening velocity (top data, *n* = 8–20), were not significantly correlated with temperature.

In a separate study, temperature dependence of force in active muscle during lengthening and shortening at different velocities was examined by applying a ramp length change of ~6% L_0_ on the plateau of an isometric tetanic contraction (Figure 6). As is well known, a ramp lengthening increased and a ramp shortening decreased the muscle tension to approximately “steady” levels and in a velocity-dependent way. Pooled data in Figure 7 shows that the isometric tension (P_0_) and the (lower) steady tension at three shortening velocities, increased with warming from 10 to 35 °C. As shown by the curves fitted to each data set, the relation between tension and reciprocal absolute temperature (1/T) was sigmoidal. However, as reported in detail in [39], the tension–temperature curve of shortening muscle was sharper, and shifted to higher temperature with increased velocity (Figure 7 legend). In contrast, the higher steady tension reached during lengthening at a given velocity was largely temperature-insensitive (within the same temperature range); in lengthening muscle, the tension–temperature curve may be shifted to lower temperatures.

The above findings basically illustrate that force generation in active muscle is endothermic and strain-sensitive. During shortening, with a faster crossbridge cycling and attached crossbridges being exposed to negative strain, temperature-sensitivity of force is more pronounced. During lengthening on the other hand, force generation is depressed as the crossbridge cycle slows in a velocity-dependent way and temperature sensitivity is less ([43,44,45] and refs therein).

## 4. Skinned Fibre Experiments

Use of skinned fibres enables one to readily alter the chemical composition of the intracellular medium around the actin–myosin contractile system. Moreover, experimental preparations can be short segments of single fibres; the preparation used in many was from rabbit psoas (a fast muscle). Such experiments on skinned fibres have shown that the force rise to a T-jump and sigmoidal increment of steady force with temperature are not seen in rigor fibres (depleted of ATP and crossbridges attached but not cycling), nor in relaxed fibres (crossbridges detached) [10,17].

**Figure 8:**
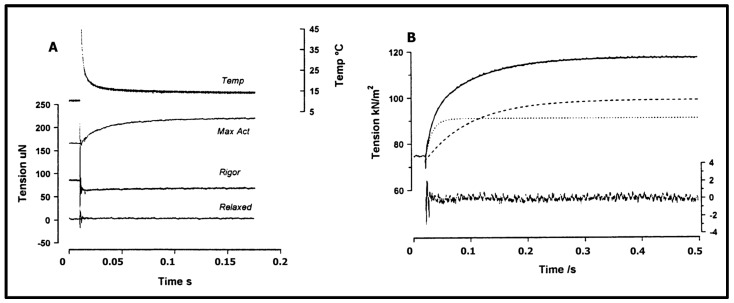
(**A**) Tension responses to a 5° T-jump from a single fast (psoas) fibre preparation at −10 °C, when it was relaxed (bottom tension trace), in rigor (middle trace), and when maximally Ca-activated (top tension trace). The T-jump was the same and a thermocouple trace is shown: from [10]. Note that the relaxed fibre tension remains unaltered, the rigor tension is decreased abruptly to a steady level, and the active tension is increased along a characteristic time course to a new steady level. (**B**) The active tension rise to T-jump could be fitted with a bi-exponential function (solid curve through the tension trace); the two exponential curves are shown separately by the dotted (phase 2b) dashed (phase 3) and the residuals after the curve fit (bottom trace).

Figure 8A shows experimental records from a muscle fibre to a T-jump from ~10 to 15 °C when in rigor, relaxed or maximally activated, from [10]. Rigor force dropped instantly with a T-jump (phase 1) and showed no recovery, and the relaxed fibre tension was unaltered. The active tension increased to a new steady level and Figure 8B shows that the rise could be resolved into two components, a faster phase 2b (endothermic force generation) and a slow phase 3), as in the intact fibre experiment (Figure 5).

Similar experiments on fibres from different muscle types (slow and cardiac) showed the same overall picture: however, under the same conditions, the speed of rise of active force to the same T-jump was slower in slow and cardiac fibres than in fast psoas fibres; at 12 °C, phase 2b rate of rat cardiac fibres was only 10% of that of fast fibre [20], perhaps, as expected from the different myosin isoform types they contain. The temperature dependence of maximally Ca-activated steady isometric force in all these fibre types also showed the sigmoidal distribution, with half-maximal force at around 10–15 °C.

### 4.1. Temperature Dependence of Steady Force and Effect of Pi and ADP

**Figure 9:**
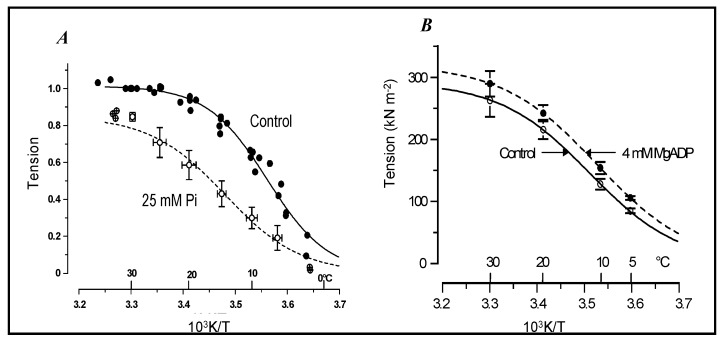
(**A**) Effect of Pi. Pooled tension values from five fibres (with maximal Ca-activation) measured at different temperatures from ~5 to 30 °C. A fibre was activated at ~5 °C, and its temperature increased by laser T-jumps and/or Peltier. Tension values were scaled to the “control” at 30 °C, and plotted against reciprocal absolute temperature. The solid curve and filled circles were from activation in the “control” solution (no added Pi). Open symbols show the mean (±SD) pooled tensions with 25 mM Pi present from two series (i.e., before and after control). Pi depresses tension, but at higher temperatures, the relative Pi-induced depression is less, and the curve is left-shifted (from Coupland et al. [13]). (**B**) Effect of ADP. Pooled tension data from 18 fibres in control solution or with 4 mM MgADP added, recorded at different temperatures (mean (±SEM)). Specific tension values (in kN m^−2^) are given for the control (open symbols) and for the fibres with 4 mM MgADP added (filled symbols). Tension is potentiated with the added ADP: the relative potentiation is less at higher temperatures and the curve is right-shifted (from Coupland et al. [46]).

A sigmoidal temperature dependence of isometric force, similar to intact fibres, is seen in maximally Ca-activated skinned fibres [21], Figure 9 from two studies (Coupland et al. [13,46]) show the effect of increased inorganic phosphate (Pi) and MgADP (Pi and MgADP are two products released during crossbridge cycling; Pi is released earlier, and ADP later in the cycle [47,48,49,50,51,52]). Figure 9A shows that active force is depressed by Pi so that the curve is shifted to higher temperatures. Figure 9B shows that MgADP increased force, and the curve is shifted to lower temperatures. It is also seen that the relative effects on tension at a given level of Pi (or ADP) is less at higher temperature (the force depression by 25 mM Pi is ~50% at ~10 °C, where as it is ~20% at physiological temperatures of ~30 °C, Figure 9A).

### 4.2. T-Jump Induced Tension Transients: Effects of Pi and ADP

**Figure 10:**
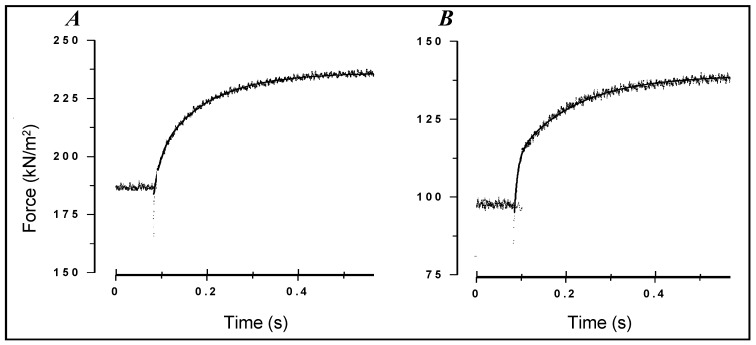
Effect of Pi. Tension response at the tension plateau at ~9 °C to a T-jump of ~3 °C. (**A**) For the control fibre (no added Pi). (**B**) The same fibre after reactivation with 12.5 mM Pi added. Each transient has been fitted with a bi-exponential curve. The extra Pi depresses steady force, but the initial T-jump transient (phase 2b) is faster. The tension rise amplitude is similar to the control (adapted from [11]).

**Figure 11:**
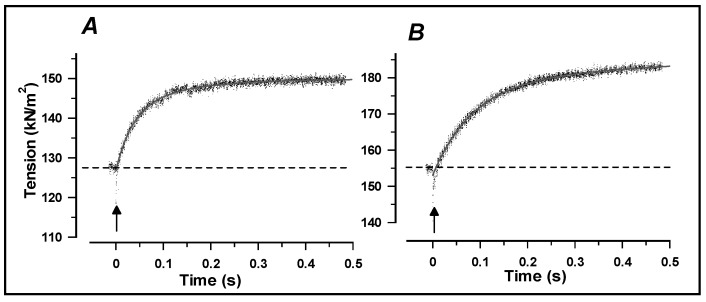
Effect of MgADP. (**A**) Tension response in the control fibre (no added MgADP), experimental design as in Figure 10. (**B**) Altered response of the same fibre reactivated in the presence of 4 mM additional MgADP. Note that the initial plateau tension level is higher, but the tension rise induced by the T-jump is slower with MgADP present; the amplitude of the rise in tension is similar (from [46]).

To define the molecular step in the ATPase cycle underlying endothermic force generation, force responses generated by standard T-jumps and at ~9 to 10 °C were examined in control, or with added Pi or MgADP. Figure 10A shows the “control” force response induced by a T-jump, and Figure 10B shows the force response from the same fibre that had been reactivated with 12.5 mM of added Pi. Compared to the control, the steady force before and after the T-jump is lower with added Pi, but the initial force rise (phase 2b) is faster with Pi. Figure 11A shows similar experiments to Figure 10, but this time, from the control and the same fibre with 4 mM MgADP added. Compared to the control, the plateau tension level is higher, but the force rise after the T-jump is slower with ADP present.

**Figure 12:**
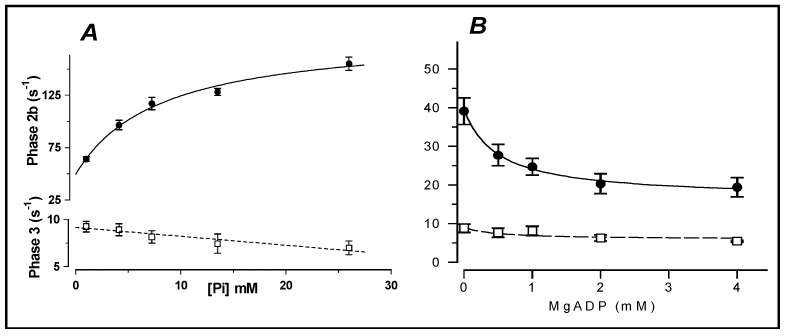
From experiments, as in Figure 10 and Figure 11 (T-jump from ~9 to 12 °C), the mean (±SEM) phase 2b rate (filled symbols), and phase 3 rate (open symbols) are shown. (**A**) Pi-dependence: the phase 2b rate increases with Pi, to a plateau, and the relation is hyperbolic (the curve fitted). Phase 3 shows minimal sensitivity to Pi (adapted from [11]). (**B**) MgADP dependence of rate: the phase 2b rate decreases with increased ADP (exhibits saturation at high ADP levels); the relation is hyperbolic. Phase 3 shows minimal sensitivity to ADP (adapted from [46]).

The different effects of Pi and ADP on the T-jump force transients are illustrated in Figure 12. The amplitude of the tension rise was much the same (not illustrated), and phase 3 was not very sensitive to Pi or ADP. On the other hand, endothermic force generation (phase 2b) became faster with increase of Pi (Figure 12A), whereas it slowed with ADP increase (Figure 12B); in both cases, the time course change reaches saturation at higher levels.

Inorganic phosphate (Pi) is released earlier in the crossbridge cycle, and the isometric plateau force is decreased with added Pi [48,53]. However, the rate of the approach to the new steady state was enhanced. Studies using different techniques have given similar results, including hydrostatic pressure-release (P-jump, [54]), sinusoidal length oscillations [55], Pi-jump [49,56], and Pi-measurement [51]. The common conclusion that came from these different studies was that Pi-release in active muscle occurs in two reversible steps. The crossbridge force generation precedes Pi-release. The results from the T-jump experiments mentioned above are consistent with this conclusion (Figure 10 and Figure 12A).

The potentiation of active tension when [MgADP] is increased may be due to binding of MgADP to AM (nucleotide-free crossbridges). This would lead to accumulation of force-bearing AM-ADP states [50,53,57,58,59] and tension increase. The tension rise induced by a T-jump was slower (Figure 11 and Figure 12B), when [MgADP] was increased; this indicates that the approach to the new steady state at the post-T-jump temperature is now slower.

### 4.3. T-Jump Effect on Force in Shortening and Lengthening Muscle

The T-jump experiments discussed above were on muscle fibres held isometrically, whereas it is known that the force that a muscle can develop changes with the velocity of filament sliding during steady muscle shortening and lengthening. At higher shortening velocities, force drops below the isometric force (P_0_), and falls to zero at the maximum shortening velocity. On the other hand, active muscle tension increases to ~2 × P_0_ as lengthening velocity increases to 1–2 L_0_ (muscle fibre lengths per second) [60,61,62]. See also the mammalian muscle data in Figure 7. Both the energy production and the acto-myosin ATPase rate increase with muscle shortening, and decrease with lengthening [63,64,65,66].

**Figure 13:**
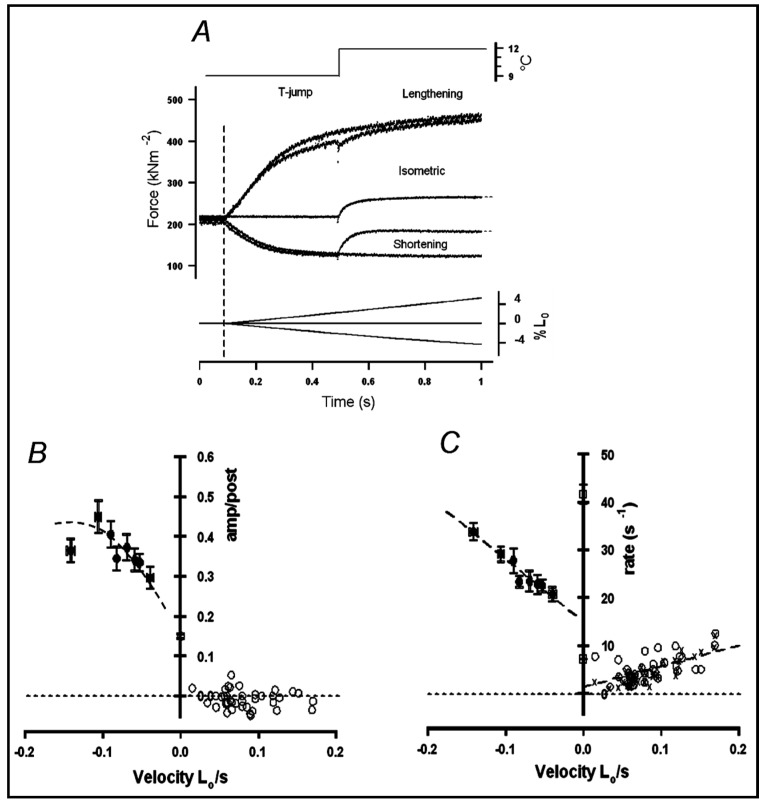
(**A**) A fibre held isometrically was maximally Ca-activated at ~9 °C and, during the tension plateau (P_0_), a T-jump of ~3 °C was applied (top panel—schematic) to obtain the “isometric” tension trace; (the bottom panel shows length records). Temperature was clamped again at ~9 °C, and the fibre lengthened at a constant velocity to obtain the “lengthening” tension traces (one without and the other with a T-jump). During lengthening, the tension rises to ~2.2 P_0_. The T-jump does not lead to a net tension increase (induces a small instantaneous tension drop, phase 1). The same procedure was repeated, but with shortening, to obtain the “shortening” tension traces. Tension drops to ~0.5 P_0_, but the T-jump induces a pronounced tension rise. The T-jump tension trace fitted well to a single exponential function. (**B**,**C**) Data from six fibres. Velocity is plotted as L_0_/s on the horizontal axis, negative for shortening, and positive for lengthening. (**B**) The amplitude (net tension change after a T-jump) is plotted (as a ratio of the post-T-jump tension). During lengthening, the tension change (open symbols, individual data) is not significantly different from zero: during shortening (filled circles) T-jump tension rises with velocity (Mean (SEM, *n* = 30) values for isometric are plotted on the ordinate). (**C**) During lengthening, the rate of tension rise from curve fit to the late part of pre-T-jump tension trace (crosses) is not significantly different (*p* > 0.1) from the post-T-jump rate (open circles). With shortening, the rate of tension rise increases with velocity (*p* < 0.001).). Two isometric rates (phase 2b and 3) are on the ordinate (adapted from [67]).

In one study [67], in addition to recording under isometric conditions as before, we also examined the T-jump response in maximally Ca-activated muscle fibres, while their force was lowered to a steady level by ramp shortening, or increased to a nearly steady level by ramp lengthening. The superimposed tension responses in Figure 13A illustrate the T-jump tension responses (middle panel) of a fibre in the three different mechanical states. As discussed before, a T-jump induces a biphasic rise in tension in an isometrically-held fibre (middle tension trace). With ramp length increases (top two tension traces), the tension rises towards a level about two-times the isometric tension; a T-jump produces no tension increment. An instantaneous but small drop in tension was sometimes seen (some thermal expansion in the fibre, [10]). During steady shortening (lower two tension traces), the tension drops to a level lower than isometric, and a T-jump produces a marked (monophasic) tension rise.

The velocity range used in this study was 0–0.2 L_0_/s and the unloaded (maximum) shortening speed at this temperature was ~1 L_0_/s; force decreased to <0.5 × P_0_ when shortening at 0.2 L_0_/s and increased to 2–3 × P_0_ when lengthening at >0.05 L_0_/s (for details, [67]).

Figure 13B shows that the amplitude of the T-jump tension rise in shortening fibres is higher than isometric, and is correlated (increased) with velocity (filled circles). There is no significant tension change induced by the by T-jump during lengthening. Figure 13C shows that the rate of tension rise induced by the T-jump during steady shortening increases with velocity. With lengthening, the rates of tension rise determined by curve fitting to the late part of the pre-T-jump tension trace (crosses) were not significantly different from the post-T-jump rates (Students *t*-test, *p* > 0.05). In isometric state, the T-jump-induced tension rise contained two components (40–50 s^−1^ and 5–10 s^−1^). Thus, the data in Figure 13 show that the tension during steady lengthening is not changed by a T-jump, whereas the tension in shortening is enhanced by a standard T-jump and in a velocity-dependent manner.

### 4.4. T-Jump Effect at Higher Shortening Velocities

**Figure 14:**
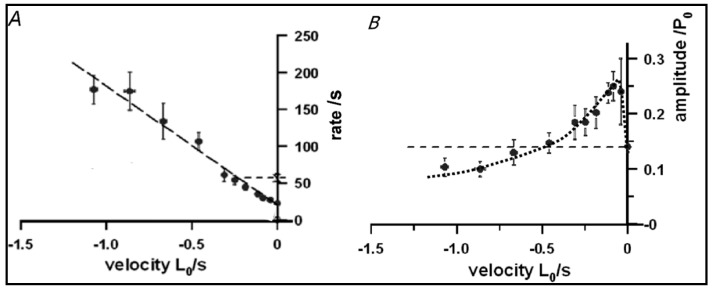
Characteristics of the T-jump-induced tension rise during steady shortening. Mean data from nine fully activated fibres where a 3–4 °C T-jump was imposed on over a wide range of shortening velocities at 8–9 °C. A single exponential curve was fitted to the post-T-jump tension rise to extract the rate and amplitude of the tension rise. The mean (±SEM, *n* = 5–18) data are plotted against shortening velocity as in Figure 13. (**A**) The rate of tension rise. Filled symbols show the rate induced tension rise produced by the T-jump, where the dashed line shows the fitted linear regression to the original data (excluding values for isometric). The isometric phase 2b from biphasic analysis was ~55/s (× on the ordinate and short-dashed horizontal line). (**B**) The amplitude of the T-jump tension rise, as a percentage of tetanic force (adapted from [68]).

On the basis of the observations in Figure 13, it was of interest to examine T-jump effect at higher shortening velocities to cover the full force–velocity curve. Such a study [68] showed that at ramp velocities approaching Vmax (~1–2 L_0_/s) at this temperature (~9 °C), a small T-jump induces a very fast tension rise. Pooled data from this study are shown in Figure 14.

As seen in Figure 13, the results show that the monophasic rate of T-jump tension rise increases linearly with shortening velocity; additionally, results in Figure 14 show that near Vmax (when force is near zero), the rate can be as high as ~200/s, or about ~4-times higher than in isometric phase 2b. The amplitude of the T-jump tension rise, when normalized to post T-jump tension, also increased as in Figure 13B. Perhaps the more interesting finding is what is displayed in Figure 14B. When the absolute increment of tension by T-jump is plotted against shortening velocity, the distribution is bi-phasic [68]; the amplitude of tension increases above isometric at lower velocities, but decreases to be below isometric (the dotted line in Figure 14B) at the higher velocities.

### 4.5. T-Jump Effect at the Onset of Ramp Shortening

In the well-known 1977 experimental study by discussing tension transients to small, rapid shortening and lengthening steps, Ford, Huxley & Simmons [69] showed that the quick tension recovery following a small L-release was also seen at the beginning of ramp shortening. This was represented by an inflection (a drop in the slope) on tension decline (Figure 29 in [69]). The X-ray diffraction studies on muscle [70,71] showed that a structural change of actin attached crossbridges occurs early in the transition from isometric to shortening. Interestingly, somewhat similar observations were made by Podolsky [72] in experiments on frog muscle, but in the initial velocity transient to sudden decrease in force level. Therefore, in a study [73] on maximally Ca-activated single muscle fibres at ~9 °C as above, we examined the temperature sensitivity of this initial tension decline by applying a T-jump coincident with onset of ramp shortening.

**Figure 15:**
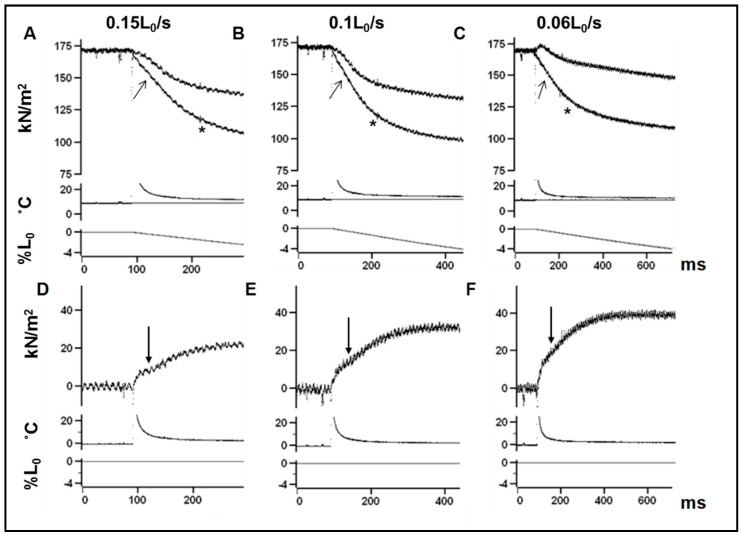
(**A**–**C**) An experiment on a maximally Ca-activated fibre at ~9 °C. A ramp shortening (lower traces) was applied at the tension plateau. Two recordings were made at each velocity: one without a T-jump (lower of the tension traces), and the other with a T-jump of ~3 °C applied at the onset of ramp shortening (middle trace is thermocouple output). The arrow and asterisk denote early (P1) and late (P2) transitions towards steady shortening state. Records show that a T-jump changes the tension decline during shortening (from the same isometric force). (**D**–**F**) For each velocity, the recording made without a T-jump was subtracted from that made with a T-jump, and the difference traces for tension (top), temperature (middle), and length (bottom) are shown (adapted from [73]).

In order to determine the T-jump effect when force is declining during ramp shortening, it was necessary to make two tension recordings at each ramp velocity, one without and the other with a T-jump, and their difference was examined. Examination of the (superimposed) tension traces with and without T-jump (upper and lower) in Figure 15A–C shows that the amplitude and time courses of the tension decline during subsequent shortening is altered by a T-jump. The difference tension traces derived from them in Figure 15D–F show that the net effect is a biphasic tension rise by a T-jump, and it is velocity sensitive.

The biphasic difference tension traces could be analysed by two-exponential curve fits to obtain the rate and amplitude of phase 2b and phase 3. Pooled data from nine experiments for the rates and amplitude showed that their distributions against shortening velocity were qualitatively similar to those in Figure 14 at steady velocities. Phase 2b and phase 3 rates were correlated and increased linearly with shortening velocity. However, the phase 2b rate increased with shortening velocity so that near Vmax (>1 L_0_/s), the rate increased to ~600/s, ~ten-fold faster than at isometric. The amplitudes (for both the phase 2b amplitude and the total, phase 2b + phase 3) at low velocities are larger than the in isometric case, and decrease to below isometric at the higher velocities. Thus, the tension increment induced by T-jump at the onset of ramp shortening also showed a biphasic dependence on velocity, as also obtained during steady shortening experiments (Figure 14).

Basically, when ramp shortening is applied to an isometrically-contracting muscle, all the attached crossbridges become increasingly negatively-strained, causing the pre-stroke crossbridges to go through the force-generating transition. If the effects of series end compliance are ignored, the tension decrease at the start of ramp shortening would be sarcomeric compliance, because there would be no time for crossbridge attachment/detachment steps to occur to an appreciable extent. The rate of tension decline will not continue at this value, due to crossbridge force generation resulting in the observed inflection. This is consistent with the interpretation given in the X-ray diffraction studies [70,71]. The fact that a T-jump enhances this (on absorption of heat) confirms that the crossbridge force generation induced by negative strain in muscle is endothermic [73].

## 5. The Acto-Myosin ATPase Cycle and Modelling/Simulation

**Scheme 1:**
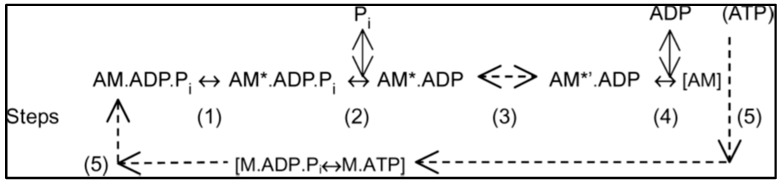
ATPase/crossbridge cycle.

A minimal, 5-step, kinetic scheme for the crossbridge/AM-ATPase cycle (Scheme 1, adapted from Lymn & Taylor [3]), can qualitatively simulate some of the findings above ([46,67]).

The step 1 is (endothermic) force generation, step 2 is Pi release/binding (similar to Dantzig et al. [49]); steps 3 and 4 represent the slow, two-step, ADP release (forward biased—see Dantzig et al. [50]). Step 5 includes all the steps after ADP release to reprime a crossbridge for the next cycle. The overall rate is low (k_+5_, ~10·s^−1^) (He et al. [34]). This unbranched kinetic scheme was solved by the matrix method, as described previously [74]. The “labelled” attached states (AM*.ADP.P_i_, AM*ADP and AM*′.ADP) are equal force-bearing states; the sum of their occupancy is taken as force.

As shown before [46], the temperature effects can be simulated by an increase of the rate constant k_+1_ (Q_10_ of 4, see Zhao & Kawai [75]) and a small increase in step k_+4_ (Q_10_ of 1.32), since ADP release itself is slightly temperature-sensitive (Siemankowski et al. [30]). As fully described in [46], such modelling could qualitatively show the following.

(1) The sigmoidal force versus temperature curve with half-maximal at ~10–12 °C for control. Within a temperature range of ~0 to 40 °C, the relation is shifted upwards (potentiated) and to slightly low temperatures with 4 mM added ADP, and shifted down (depressed) and to higher temperatures with increased Pi.
(2) A resetting of k_+1_ to a higher value simulated tension responses induced by a T-jump [74]. With a T-jump, the tension rise was faster with Pi, but slower with ADP.
(3) A shortening was simulated by increasing k_+4_, and during such simulated steady shortening, a T-jump tension response was faster at higher velocity and, at a given velocity, T-jump tension response became faster at higher temperatures—as experimentally found [67,68].

It is relevant to note that the modelling and simulations referred to above were simplistic, used a minimal unbranched actin–myosin–ATPase pathway. To fully address the mechanics and energetics of active muscle, more detailed mechanokinetic modelling would be necessary, as in [33,76,77,78]; but such modelling has not been extended to examine temperature-effect and temperature-jump findings in muscle. Kinetic modelling was useful to gain a qualitative picture, and it seems that an unbranched kinetic cycle, as above, can account for the main observations. However, the very fast initial tension recovery, phase 2a, seen after length-release, is not considered; this may be a consequence of viscoelasticity in the filament compliance, as suggested by Davis [42]. Also, the negative strain-sensitivity of the endothermic force generation step, such as in Figure 15, is not identified. Furthermore, studies on myosin–ATPase in solution [79,80,81] have shown that the ATP cleavage step (i.e., in detached crossbridges in fibres) is endothermic, and that is included in the scheme above. As Offer & Ranatunga [82] examined previously, the experimental findings that sarcomeric filaments in muscle are not only compliant, but also that their compliance may be non-linear [83,84,85,86], needs to be accommodated for a fuller picture.

## 6. Discussion

Findings from various temperature studies on intact and skinned mammalian muscle fibres, briefly referred to in this review, provide some useful information relating to the processes and mechanisms of muscle contraction and function. Although not fully understood, a general issue some observations raise is that the underlying mechanism of a contractile event (maximum shortening velocity) in muscle may not be the same at different temperature ranges, as mentioned in relation to Figure 1 and Figure 3. Perhaps the more important observation from the studies is that in active muscle, force is endothermic, and that force rises with temperature, upon absorption of heat. This is largely due to the force generation by an attached crossbridge state itself being temperature-sensitive. The rapid T-jump results show that the step is before phosphate release in a linear acto-myosin ATPase pathway. Also, it is strain sensitive (enhanced during shortening and depressed during lengthening). Additionally, observations on the T-jump effect at the onset of ramp shortening (Figure 15) suggests that tension recovery transient after L-release [16] also has this endothermic step.

### 6.1. Comparison with Different Perturbations

Different rapid perturbations have been used to examine the underlying mechanism of force generation in active muscle. The notion that the force generation step is prior to Pi-release (a transition between two AM.ADP.P_i_ states) is proposed in several studies using different perturbation techniques, such as hydrostatic pressure-release (P-jump, [54]), sinusoidal length oscillation [55], a Pi-jump [49,56] and Pi-measurement [51]. T-jump experiments (Figure 10), and other studies [9,42,75], show that this force generation is endothermic (and entropy driven).

In the interpretation of the original experiments and formulations by Huxley & Simmons [16], the quick recovery of tension after a rapid small length-release step is thought to represent the crossbridge power stroke and muscle force generation. The quick tension recovery can be separated into two components [19], labelled as (fast) phase 2a and slower, phase 2b; phase 2b is as after T-jump. By extrapolation, the rate of the phase 2b recovery corresponding to the isometric point in a length step versus rate of tension recovery plot in experiments on rabbit psoas fibres at ~10 °C is ~40–60 s^−1^ [15,46], similar to the speed of force rise after a T-jump. Also, tension recovery after length step is faster with P_i_ [15,87] and slower with MgADP, [46], as in T-jump tension rise. Hence, tension recovery after a length step has a homologous component to the force rise after a T-jump; this was first noted by Davis & Harrington [19]; see also [42]. Interestingly, experiments of Gilbert & Ford [88] on frog muscle, showed that quick tension recovery from length-release is associated with heat absorption (i.e., is endothermic). In addition, after a shortening step, the rate of recovery of phase 2 tension is temperature sensitive (Q_10_ of 2–3, [89]). There is greater temperature sensitivity for phase 2b than there is for the phase 2a component of recovery [9,19]. Thus, like the force rise due to a T-jump, phase 2b tension recovery after a shortening step is an endothermic process. The (fast) phase 2a kinetics after length release is probably due to some non-crossbridge viscoelasticity within muscle fibres, as suggested in several studies [19,42,90]. Interestingly, an initial fast tension recovery after stretch, as after L-release, was seen in model simulations of tension responses to length perturbations [91].

It is relevant to note that, since the time when Huxley and Simmons [16] proposed their ideas and supported these with experimental detail [69], our understanding of sarcomere elasticity and sarcomere mechanics has changed. Thus, it is now clear that sarcomeric compliance is not just in attached crossbridges, but also in thick and thin filaments (refs in [78,82]), compliance may be non-linear, and their effects could be complex [82,83,84,85,86]. Additionally, there have been many experimental studies using a variety of techniques in different laboratories [17,37,38,42,55,92,93], which all indicated that the active force-generation process in muscle is endothermic. However, its relationship to force recovery induced by a length release, as in the Huxley et al. [16,69] experiments, has remained unclear [12]. Indeed, Kawai and Halvorson [55], Bershitsky et al. [94,95], Piazzesi et al. [89], and Davis and Epstein [9] all suggested that the endothermic force-generating process and the step-release-induced force recovery represent different steps in the muscle crossbridge cycle. Also, Huxley [96], Ferenczi et al. [97], and Woledge et al. [98] suggested that there is a temperature-sensitive step in a parallel attached pathway. By contrast, a look back on previous experiments suggests that strain-sensitive crossbridge force generation is also temperature sensitive (endothermic).

Developing a model to simulate the steady state force–velocity data, L-step force transients, crossbridge stiffness, and energetics in active frog muscle, Offer and Ranatunga [78] found that two force generation steps (of similar magnitude) are essential in the crossbridge cycle; the cycle used was simple, linear, and unbranched, as in the scheme above. This contrasted with other complex schemes proposed from X-ray studies of shortening muscle of Huxley et al. [99] that suggested several molecular steps would be necessary to complete a working stroke of a crossbridge. Interestingly, when the model in [78] with two force-generating steps was used to simulate the basic effects of temperature [100], it was found that the strongly endothermic ATP hydrolysis and crossbridge attachment steps, as found in biochemical studies, contribute to the increase of tension in isometric and shortening muscle; however, the first force-generation step was also endothermic (as considered here). A force rise on release of hydrostatic pressure (volume increase), has been reported in some experiments on skinned rabbit fibres [54] and on intact frog fibres [14], and heat-absorbing force development could account for such an effect.

### 6.2. Structural Mechanism of Force Generation and Some Other Issues

As presented in the review by Geeves & Holmes [101], a change in crossbridge structure pulling on the lever arm is considered to be a possible mechanism of active force development. Such a process is unlikely to be endothermic; also, whether such a process—without a change in its attachment to thick filament—can generate much force remains unclear [4]. Changes in attachments of crossbridge states (non-stereospecific to stereospecific, hydrophilic to hydrophobic, etc.) have been suggested in some studies [75,79,97] to account for heat absorbing (endothermic) force. Looking back on other ideas, Davis, Harrington group [19,37,42,102] have discussed several different mechanisms to account for endothermic force in muscle. Davis & Epstein [9] proposed that an unfolding within the crossbridge secondary/tertiary structure of crossbridge might cause force generation. Furthermore, they proposed an interesting idea that the forward rate of force generation step is increased, but the reverse rate is decreased with an increase of temperature. In a recent review, Sugi [4] has re-examined the possible importance of a change in crossbridge attachment (subfragment-2) during force generation. It seems that specific experimental details of an endothermic structural mechanism (with a volume increase) for crossbridge force generation in muscle that can account also for its coupling to acto-myosin cycle are still lacking.

For completeness, it is relevant to note that this brief review examined (looked back on) the experimental findings with respect to temperature effects on force of maximally activated muscles and muscle fibres. Thus, in experiments on intact muscle, the temperature effects were with respect to (fully fused) tetanic contractions and in skinned fibre experiments, fibres were maximally Ca-activated. The temperature effects on submaximal contractions could be more complex, since Ca-sensitivity of actin filament activation changes with temperature [103], and T-jump effects also show complexity both in skinned fibres [17] and in intact fibres [104]. Moreover, pH change can have some effects [105], although the pH buffers used in these experiments were relatively resistant, and also, T-jump amplitude small. Understanding of these issues on temperature effects on submaximal contractions is important, since in situ muscles can function at different activation levels, but they need to be examined in relation to excitation–contraction coupling, in addition to crossbridge cycle.

## Abbreviations

A	actin
ADP	adenosine diphosphate
ATP	adenosine triphosphate
L_0_	optimal muscle fibre length
M	myosin
P1, P2	various tensions
P_0_	maximal isometric (tetanic) tension
Pi	inorganic phosphate
Q10	temperature coefficient
V	velocity of shortening
Vmax	maximum
